# Humoral Immune Responses to EGFR-Derived Peptides Predict Progression-Free and Overall Survival of Non-Small Cell Lung Cancer Patients Receiving Gefitinib

**DOI:** 10.1371/journal.pone.0086667

**Published:** 2014-01-31

**Authors:** Koichi Azuma, Nobukazu Komatsu, Satoshi Hattori, Satoko Matsueda, Akihiko Kawahara, Tetsuro Sasada, Kyogo Itoh, Tomoaki Hoshino

**Affiliations:** 1 Division of Respirology, Neurology, and Rheumatology, Department of Internal Medicine, Kurume University School of Medicine, Kurume, Fukuoka, Japan; 2 Department of Immunology, Kurume University School of Medicine, Kurume, Fukuoka, Japan; 3 Biostatistics Center, Kurume University, Kurume, Fukuoka, Japan; 4 Department of Diagnostic Pathology, Kurume University Hospital, Kurume, Fukuoka, Japan; University of Barcelona, Spain

## Abstract

Somatic mutations in the epidermal growth factor receptor (EGFR) gene are associated with clinical response to EGFR tyrosine kinase inhibitors (TKIs), such as gefitinib, in patients with non-small cell lung cancer (NSCLC). However, humoral immune responses to EGFR in NSCLC patients have not been well studied. In this study, we investigated the clinical significance of immunoglobulin G (IgG) responses to EGFR-derived peptides in NSCLC patients receiving gefitinib. Plasma IgG titers to each of 60 different EGFR-derived 20-mer peptides were measured by the Luminex system in 42 NSCLC patients receiving gefitinib therapy. The relationships between the peptide-specific IgG titers and presence of EGFR mutations or patient survival were evaluated statistically.

IgG titers against the egfr_481–500, egfr_721–740, and egfr_741–760 peptides were significantly higher in patients with exon 21 mutation than in those without it. On the other hand, IgG titers against the egfr_841–860 and egfr_1001–1020 peptides were significantly lower and higher, respectively, in patients with deletion in exon 19. Multivariate Cox regression analysis showed that IgG responses to egfr_41_ 60, egfr_61_80 and egfr_481_500 were significantly prognostic for progression-free survival independent of other clinicopathological characteristics, whereas those to the egfr_41_60 and egfr_481_500 peptides were significantly prognostic for overall survival. Detection of IgG responses to EGFR-derived peptides may be a promising method for prognostication of NSCLC patients receiving gefitinib. Our results may provide new insight for better understanding of humoral responses to EGFR in NSCLC patients.

## Introduction

Lung cancer is the leading cause of cancer death worldwide [1]. The epidermal growth factor receptor (EGFR), one of the most studied tyrosine kinase receptors, is a prototypic cell-surface receptor that can be targeted by drugs against lung cancer. The EGFR family is known to play an important role in the regulation of cell proliferation, differentiation, and migration [2]. Somatic mutations in the EGFR gene have been identified as a major determinant of the clinical response to treatment with EGFR tyrosine kinase inhibitors (TKIs), such as gefitinib and erlotinib, in patients with non-small cell lung cancer (NSCLC). Most of the EGFR mutations occur in exons 19 to 21, which encode the tyrosine kinase domain of the receptor. Deletions in exon 19 (such as delE746-A750) and the L858R point mutation in exon 21 are the commonest mutations found in NSCLC, accounting for about 90% of all EGFR mutations. These mutations are found more frequently in female patients, in individuals who have never smoked, and in patients of East Asian ethnicity [3]–[5]. Prospective clinical trials of EGFR-TKI treatment in NSCLC patients with *EGFR* mutations have demonstrated remarkable response rates in the order of 80% [6]–[8].

Previously, we have developed personalized peptide vaccination (PPV) as a novel modality for cancer therapy, in which vaccine antigens are selected on the basis of pre-existing immune responses against tumor-associated antigens (TAA) [9]–[13]. We reported that immunoglobulin G (IgG) responses to TAA-derived CTL epitope peptides were well correlated with overall survival (OS) in patients with advanced cancer undergoing PPV [14], [15]. These results suggested that humoral immune responses against TAA-derived peptides might significantly impact the clinical course of cancer patients. However, there is little information regarding the clinical significance of humoral immune responses to EGFR-derived peptides in NSCLC patients.

Recently, novel high-throughput technologies have been developed for discovering biomarkers that clearly reflect clinical outcomes and/or responses to treatment in patients with cancer [16]–[21]. In the present study, we employed the high-throughput Luminex suspension array system to measure IgG responses to EGFR-derived peptides in patients with NSCLC. Here we report for the first time that IgG responses to some EGFR-derived peptides are detectable in NSCLC patients, and that they could be potentially useful predictors of progression-free (PFS) and OS in NSCLC patients receiving gefitinib.

## Materials and Methods

### Patients, treatments, and sample collection

We enrolled 42 NSCLC patients treated with gefitinib between 2006 January and 2008 December at a single institution (Kurume University Hospital, Kurume, Japan). Details of the patients’ clinical characteristics, including age, sex, histology, smoking status, performance status (PS), stage, treatment response, and type of *EGFR* mutations were obtained from chart reviews by an independent reviewer who was unaware of the clinical courses (Table 1). All of the patients had advanced NSCLC and received gefitinib (250 mg) orally once a day. Tumor response was examined by computed tomography (CT) and was evaluated according to the Response Evaluation Criteria in Solid Tumors (RECIST). Response was confirmed at least 4 weeks (for a complete or partial response) or 6 weeks (for stable disease) after it was first documented. Plasma samples were collected from the patients before gefitinib treatment and frozen at –80°C until use. Plasma was also collected from healthy donors (HD) (n = 20, 59+/–11years, Male = 8, Female n = 12). The present study complied with the provisions of the Declaration of Helsinki, and was approved by the Institutional Review Board of Kurume University. Written Informed consent was obtained from all subjects.

**Table 1:** Patients' characteristics.

Characteristics		Number
Age (years)		
	Median	63.5
	Range	38–82
Gender		
	Male	17
	Female	25
Histology		
	Adenocarcinoma	38
	Squamous cell carcinoma	4
Smoking status		
	Never smoker	24
	Smoker	18
Perfomance status		
	0	34
	1	4
	2	4
c-Stage		
	StageIIIB	4
	StageIV or reccurent	38
EGFR mutation		
	746DEL	8
	L858R	13
	Negative	21
Treatment response		
	Partial response (PR)	19
	Stable disease (SD)	14
	Progressive disease (PD)	9

### EGFR mutation analysis

Mutations of the EGFR gene were examined in exons 19 (E746-A750del) and 21 (L858R) by peptide nucleic acid-locked nucleic acid (PNA-LNA) PCR clamp, as described previously [22]. In brief, genomic DNA was purified from paraffin-embedded tissues using a QIAamp DNA Micro kit (Qiagen, Inc., Valencia, CA). The PCR primers employed were synthesized by Invitrogen (Carlsbad, CA). PNA clamp primers and LNA mutant probes were purchased from FASMEC (Kanagawa, Japan) and IDT (Coralville, IA), respectively. PNA-LNA PCR clamp was performed using a SDS-7500 System (Applied Biosystems, Foster City, CA).

### Peptides and measurement of IgG titers against peptides derived from EGFR

**Figure 1:**
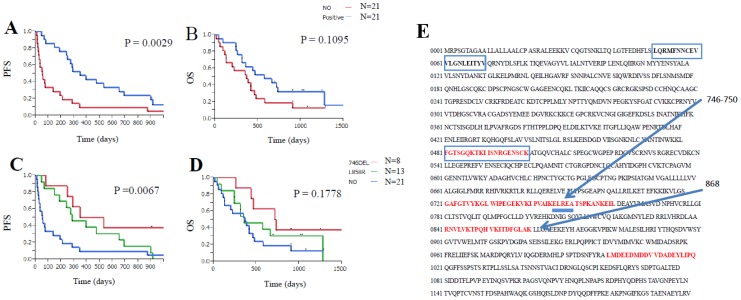
Kaplan-Meier analysis of PFS and OS in NSCLC patients receiving gefitinib treatment. Log-rank test revealed that gefitinib treatment significantly prolonged PFS (A), but not OS (B), in NSCLC patients with EGFR mutations. Significant differences in PFS (C), but not in OS (D), between patients with and without EGFR mutations were also apparent for mutations in both EGFR exon 19 (E746-A750del) and exon 21 (L858R). (E) Sixty different 20-mer peptides were designed from the amino acid sequence of EGFR protein. The sequences shown in red (egfr_481–500, egfr_721–740, egfr_741–760,egfr_841–860, and egfr_1001–1020) represent the peptides exhibiting specific IgG responses that were correlated with EGFR mutations. The sequences shown in blue (egfr_41_ 60, egfr_61_80, and egfr_481_500) represent the peptides exhibiting specific IgG responses that were correlated with PFS.

Sixty different non-overlapping 20-mer peptides were designed from the sequence of the EGFR protein and synthesized by Sigma Aldrich (St. Louis, MO), as shown in Figure 1E. The peptides were dissolved in DMSO as reported previously [23]. The IgG titers specific to each of the peptides were measured using a multiplex bead suspension array on the Luminex system, as reported previously [24]. In brief, 100 µl of diluted plasma was incubated with xMAP beads (Luminex Corp., Austin, TX), which were coated with the EGFR-derived peptides, in a 96-well filter plate (MABVN1250; Millipore Corp., Bedford, MA) for 2 h at room temperature on a plate shaker. The plate was then washed and incubated with 100 µl of biotin-conjugated goat anti-human IgG (BA-3080; Vector Laboratories, Burlingame, CA) for 1 h at room temperature on a plate shaker. After washing, 100 µl of streptavidin-PE was added to the wells, and the plate was incubated for 30 min at room temperature on a plate shaker. The beads were washed three times, followed by addition of 100 µl of PBS to each well. The fluorescence intensity in 50 µl of each sample was examined using the Luminex system. The peptide-specific IgG titers were estimated in terms of fluorescence intensity and expressed as fluorescence intensity units (FIU), as reported previously [24]. The cut-off level was set at 10 FIU because the FIU curves obtained from the sample dilution assays were linear from 10 to 10,000 FIU (data not shown).

### Statistical analysis

To examine whether IgG titers against each of the 60 different peptides were associated with EGFR mutation status, their median values were compared among patients with EGFR mutations (delE746-A750 and L858R) and the wild-type EGFR using the Wilcoxon rank-sum test. PFS was calculated from the date of initiation of gefitinib treatment until either the date of disease progression or the date of last contact. OS was defined as the period from the date of initiation of gefitinib treatment until the date of death due to any cause, or to the date of last contact. To examine whether IgG titers against each of the 60 different peptides were associated with PFS or OS, we applied the Cox proportional hazards model with anti-peptide IgG titers, mutation status, smoking status, gender, and PS as explanatory variables. We also examined whether they were associated with the tumor response using logistic regression, where CR and PR were regarded as responses. Since 60 different peptides were examined, a severe multiplicity issue existed in this study. Therefore, we identified peptides that were significantly associated with PFS, OS, or tumor response by controlling the false discovery rate (FDR) at the 5% level.

In this study, it was considerably challenging to identify the anti-peptide IgG responses that would be useful for prognostication than simply using clinicopathological characteristics alone. Since the number of peptides exceeded the number of patients, the standard multivariate Cox regression (multiple regression) could not be employed. To avoid influential observations, anti-peptide IgG was log-transformed (anti-peptide IgG +1), and also standardized with zero-mean and unit standard deviation. We applied the Cox regression with a lasso-type penalty [25], [26], which has recently been reported to be useful for analysis of high-dimensional data. Since a notable feature of the lasso method is its sparsity, regression coefficients for anti-peptide IgG responses not associated with PFS (OS) could be estimated as zero. Based on this feature, we identified a few peptides that might be useful for prognostication. To examine whether or not IgG responses to the identified peptides were, in fact, useful for prognostication, we applied Cox regression analysis and time-dependent ROC analysis [27]. Areas under the ROC curve (AUCs) were estimated for risk scores by Cox regression with clinicopathological characteristics alone and also with both anti-peptide IgG responses and clinicopathological characteristics. They were compared by testing the equality of AUCs by calculating a bootstrap p-value for 1000 replicates. Statistical analysis was performed with R version 2.13 and SAS version 9.3 software (SAS Institute, Cary, NC).

## Results

### Patient characteristics and survival analysis

The clinical characteristics of the 42 patients are shown in Table 1. Twenty-five patients (60%) were female and 24 (57%) had never smoked; the median age of the patients overall was 63.5 years (range, 38 to 82 years). Thirty-eight patients (90%) had adenocarcinoma, 34 (81%) had a good performance status (Eastern Cooperative Oncology Group 0), and 15 (36%) received gefitinib as first-line chemotherapy. With regard to the type of *EGFR* mutation, 8 patients (19%) had deletions in exon 19, 13 (31%) had the L858R missense mutation in exon 21, and 21 (50%) had the wild-type *EGFR*.

At the time of analysis, the median follow-up period was 418 days (range, 16 to 1532 days). The median PFS was 201 days (range, 11 to 1379 days), and the median OS was 418 days (range, 16 to 1532 days). Kaplan-Meier analysis of PFS and OS after the start of gefitinib treatment is shown in Figure 1. The log-rank test revealed that gefitinib treatment resulted in a significantly longer PFS in patients with EGFR mutations than in those without them (median of 347 versus 54 days, *P*  = 0.0029) (Fig. 1A), whereas there was no significant difference in OS between the two groups of patients (median of 575 versus 368 days, respectively, *P*  =  0.1095) (Fig. 1B). The differences in PFS, but not OS, between patients with mutations and those with the wild-type EGFR were apparent for both types of EGFR mutation (Fig. 1C and 1D).

### Correlation between IgG titers against EGFR-derived peptides and EGFR mutations in NSCLC patients treated with gefitinib

We examined IgG titers against each of 60 different peptides in plasma samples from NSCLC patients using the Luminex system. We analyzed whether the peptide-specific IgG titers were correlated with the presence of EGFR mutations, and found that IgG titers specific to the egfr_481–500, egfr_721–740, and egfr_741–760 peptides were significantly higher in patients with exon21 mutation than in those without it (P = 0.017 for egfr_481–500; P = 0.036 for egfr_721–740; P = 0.007 for egfr_741–760) (Table S1). For these three peptides, the median values of peptide-specific IgG titers in patients with exon 21 mutation were about double those in patients without exon 21 mutation (Table S1). On the other hand, the titer of IgG specific to the egfr_841–860 peptide was significantly lower in patients with deletion in exon 19 than in those without it (P = 0.047), whereas the titer of IgG specific to the egfr_1001–1020 peptide was significantly higher in those with deletion in exon 19 (Table S1). IgG responses to other peptides showed no significant correlation with EGFR mutations.

### Relationship between titers of IgG against EGFR-derived peptides and survival in NSCLC patients treated with gefitinib

We further investigated whether the peptide-specific IgG titers were well correlated with PFS or OS in NSCLC patients receiving gefitinib treatment. In the Cox regression, IgG responses against 38 and 32 EGFR-derived peptides showed p-values of less than 5% for PFS and OS, respectively. When FDR was controlled at the 5% level, IgG responses against 35 and 20 peptides were identified as significant for PFS and OS, respectively ( Table S2). We also examined whether the IgG titers against each peptide were associated with tumor response (CR or PR). Logistic regression analysis indicated that there were no peptide-specific IgG responses associated with tumor response (data not shown).

### Identification of peptide-specific IgG responses useful for prognostication

As shown above, IgG responses to many of the EGFR-derived peptides were significantly associated with PFS and/or OS. Since many pairs of peptides were moderately or strongly correlated (data not shown), it was suggested that measurement of IgG titers against relatively small numbers of peptides might be sufficiently prognostic. By Cox regression with the lasso penalty, IgG titers against the egfr_41_60, egfr_61_80, and egfr_481_500 peptides had relatively large effects on PFS (Figure S1A). We employed the IgG titers against these three peptides for constructing a prediction rule for PFS. As shown in Table 2A, Cox regression adjusting for possible confounding factors, including PS, age, gender and smoking status, demonstrated that all of the IgG responses against the egfr_41_60, egfr_61_80, and egfr_481_500 peptides were significantly prognostic and independent of any clinicopathological characteristics (P = 0.001, P = 0.020, and P = 0.028, respectively).

**Table 2:** Cox regression analysis of PFS for NSCLC patients.

factor	P-value	HR	95%CI	
mutation (Mutant/Wild-type)	0.000	0.17	0.07	0.43
egfr_481_500	0.028	0.59	0.37	0.94
egfr_61_80	0.020	0.54	0.32	0.91
egfr_41_60	0.001	0.24	0.10	0.56
sex (F/M)	0.929	1.11	0.11	11.17
ps (1–3/0)	0.105	2.18	0.85	5.60
smoke (Smoker/Never)	0.913	1.13	0.12	10.83
age	0.101	1.03	0.99	1.08
factor	P-value	HR	95%CI	
mutation (Mutant/Wild-type)	0.331	0.67	0.30	1.50
egfr_481_500	0.027	0.63	0.42	0.95
egfr_41_60	0.018	0.39	0.18	0.85
sex (F/M)	0.472	0.43	0.04	4.35
ps (1–3/0)	0.199	1.82	0.73	4.54
smoke (Smoker/Never)	0.632	0.58	0.06	5.47
age	0.540	1.01	0.97	1.05

By Cox regression with the lasso penalty, IgG titers against the egfr_41_60, egfr_481_500, and egfr_881_900 peptides were shown to have relatively large effects on OS ( Figure S1B). Since IgG titers against the egfr_41_60 and egfr_881_900 peptides were strongly associated (Spearman’s rank correlation coefficient 0.71; P<0.001), we employed only the titers of IgG against egfr_41_60 and egfr_481_500 for constructing a prediction rule for OS. As shown in Table 2B, Cox regression showed that the IgG responses to both peptides were significantly prognostic, independent of any clinicopathological characteristics (P = 0.018 for egfr_41_60 and P = 0.027 for egfr_481_500).

**Figure 2:**
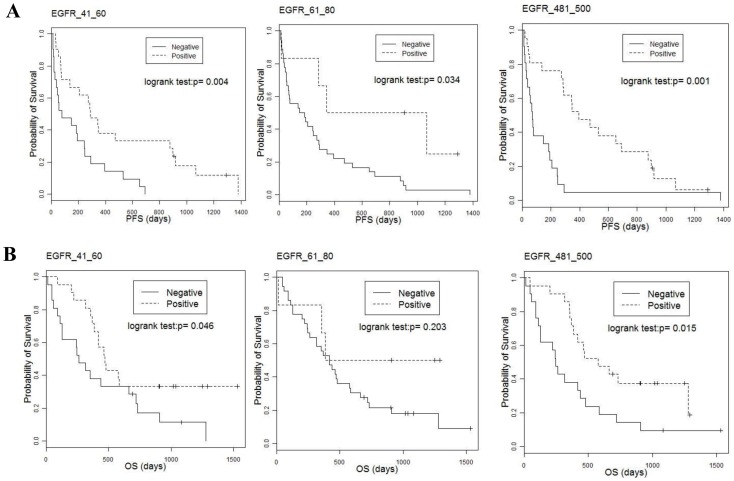
Kaplan-Meier analysis of PFS and OS after stratification by IgG titer against selected EGFR-derived peptides in NSCLC patients receiving gefitinib treatment. Kaplan-Meier plots of PFS (A) and OS (B) in patients showing higher and lower IgG titers against the selected peptides, egfr_41_ 60, egfr_61_80, and egfr_481_500, are shown. Lower and higher IgG titers were defined by their median values.

**Figure 3:**
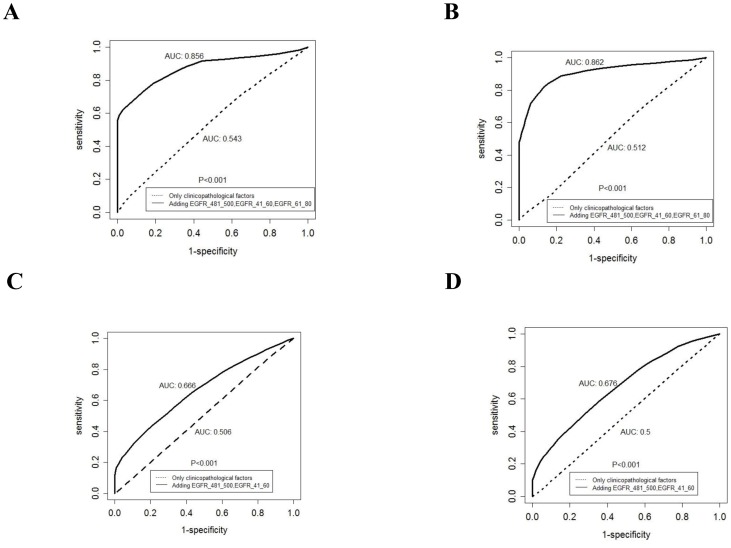
ROC curves for PFS and OS. The ROC curves for 1-year (A) and 2-year (B) PFS of the risk score estimated by Cox regression indicate a substantially improved correlation with 1-year and 2-year PFS. The ROC curves for 1-year (C) and 2-year (D) OS are also shown.

Kaplan-Meier plots of PFS and OS by stratification with IgG titers to the selected peptides are shown in Figure 2A and Figure 2B, in order to grasp their marginal effects without adjusting for clinicopathological characteristics. Using time-dependent ROC analysis, we also examined whether or not adding peptide-specific IgG titers to clinicopathological characteristics improved the accuracy of prognostication. Figures 3A and 3B show the ROC curves for 1 year and 2 years of the risk score estimated by the Cox regression given in Table 2A (for PFS) and Table 2B (for OS) with peptide-specific IgG titers and clinicopathological characteristcs and those with the latter alone. The ROC curves indicated that addition of peptide-specific IgG titers to the clinicopathological characteristics led to substantial improvement in the ability to predict PFS at 1 year and 2 years (P<0.001 by comparison of AUCs). AUCs of the time-dependent ROC for the 1-year and 2-year OS were also significantly increased by adding the peptide-specific IgG titers in comparison with clinicopathological characteristics alone (P<0.001) (Fig.3C and 3D). These findings suggested that adding peptide-specific IgG titers to the clinicopathological characteristics might lead to more accurate prognostication of both PFS and OS.

## Discussion

Recent advances in molecular oncology have dramatically improved our understanding of the growth and survival pathways of NSCLC. For example, EGFR, a member of the HER or Erb-B family of receptor tyrosine kinases, is implicated in the development and progression of NSCLC. EGFR consists of an extracellular ligand-binding domain, a transmembrane region, and a multifunctional cytoplasmic tail with integral kinase activity [2], [28]–[30]. EGF is a secreted growth factor whose binding to EGFR induces structural changes, leading to receptor homodimer formation, followed by an increase of EGFR kinase activity and subsequent phosphorylation of the intracellular domain [2], [28]–[30]. The most frequently observed mutation in EGFR is the substitution L858R in the activating loop (A-loop), or deletion of eight residues in the region spanning residues 746–759, extending from the beta3 strand to the alphaC helix in the N-lobe of the kinase domain [2]–[5], [28]–[30]. In this study, we found that the IgG responses to the peptides egfr_481–500, egfr_721–740, and egfr_741–760 were significantly higher in patients with exon 21 mutation. On the other hand, the IgG responses to the egfr_841–860 and egfr_1001–1020 peptides were significantly lower and higher, respectively, in patients with deletion in exon 19. Interestingly, egfr_721–740, egfr_741–760, and egfr_841–860 are located in the ATP-binding domain, which encodes the tyrosine kinase domain of the receptor, and humoral immune responses to these sequences were correlated with the presence of activating EGFR mutations, such as L858R or 746DEL. On the other hand, egfr_1001–1020 is located in the regulatory domain in the C-terminal tail, which can increase autophosphorylation of EGFR [31]. Previous studies have reported that this domain interacts extensively with both the C-lobe and N-lobe of the kinase domain [32]–[33]. These findings suggest that the sequences related to tyrosine kinase activity in EGFR might be immunogenic in patients with EGFR mutations, although further study is needed to clarify the mechanisms of the increased IgG responses to these sequences.

The development of rapid and precise diagnostic techniques for detecting EGFR mutations is particularly important for devising personalized therapeutics for NSCLC patients with activating EGFR mutations. Several highly sensitive methods for detection of EGFR mutations in tissue specimens have been reported [22], [37], but sample collection for these methods requires invasive procedures, such as transbronchial biopsy or pleural puncture. In contrast, the present results suggest that screening of the IgG responses to EGFR-derived peptides in peripheral blood might be feasible for detecting EGFR mutations. Detection of humoral responses against EGFR-derived peptides using the Luminex suspension array system is simple and non-invasive. In particular, this method may be useful for patients with NSCLC, whose tumor tissues are difficult to obtain for detailed pathological and molecular characterization.

We further investigated whether IgG responses against EGFR-derived peptides could be predictive of PFS and OS in NSCLC patients receiving gefitinib. We found that the IgG responses against the peptides egfr_41_ 60, egfr_61_80 and egfr_481_500 had large effects on PFS, and that those against egfr_41_60 and egfr_481_500 had large effects on OS. Interestingly, all of these sequences are located in the extracellular domain. Binding of EGF occurs within the amino-terminal 622-amino-acid extracellular domain, which consists of four domains, I-IV, of EGFR. Recently, structural data have demonstrated how anti-EGFR antibodies inhibit signal transduction from EGFR. For example, the egfr_481_500 sequence belongs to extracellular domain III, where anti-EGFR antibodies, such as cetuximab, nimotuzumab, and matuzumab, are known to bind and block the binding of EGF to EGFR [34]-[36]. Although the reasons why IgG responses to these peptides might impact on survival are not fully understood, one possible explanation is that IgG against the extracellular domain might affect signal transduction from EGFR.

We examined plasma from 20 healthy donors (HD) using the Luminex system to detect the antibodies against egfr_41_ 60, egfr_61_80 and egfr_481_500 peptides. We found that the titers of antibodies against egfr_41_60, egfr_61_80, and egfr_481_500 in plasma were not significantly different between NSCLC patients and HD (data not shown). This result is consistent with our previous finding that antibodies against cytotoxic T lymphocytes (CTL) epitope peptides derived from many of tumor-associated antigens were detected as positive in HD, and that their titers were not significantly different between cancer patients and HD [38]. This finding suggested that humoral immune responses to EGFR could be widely detectable not only in cancer patients but also in HD, since this molecule is ubiquitously expressed not only cancer tissues but also in normal tissues.

In conclusion, the present study has demonstrated that detection of humoral immune responses to EGFR-derived peptides in plasma using the Luminex suspension array system may be a promising method for not only detecting the presence of EGFR mutations but also the prognostication of NSCLC patients receiving EGFR-TKIs. These results may provide new insight for better understanding of the humoral immune responses to EGFR in NSCLC patients. Since the main drawback of this study was its small sample size, a further prospective study is now underway to confirm the findings in larger cohorts. In addition, it will be necessary to clarify the clinical applicability of our findings to personalized treatment for NSCLC patients.

## Supporting Information

Figure S1 **Solution path of the Cox regresson with a lasso penalty for PFS (A) and OS(B). (A)**By Cox regression with the lasso penalty, IgG titers against the egfr_41_60, egfr_61_80, and egfr_481_500 peptides had relatively large effects on PFS. **(B)** By Cox regression with the lasso penalty, IgG titers against the egfr_41_60, egfr_481_500, and egfr_881_900 peptides were shown to have relatively large effects on OS.
(TIF)

Table S1 **Correlation between EGFR mutation and expression of peptide in NSCLC patients.** We examined IgG titers against each of 60 different peptides in plasma samples from NSCLC patients using the Luminex system. We found that IgG titers specific to the egfr_481–500, egfr_721–740, and egfr_741–760 peptides were significantly higher in patients with exon21 mutation than in those without it. On the other hand, the titer of IgG specific to the egfr_841–860 peptide was significantly lower in patients with deletion in exon 19 than in those without it (P = 0.047), whereas the titer of IgG specific to the egfr_1001–1020 peptide was significantly higher in those with deletion in exon 19.
(XLSX)

Table S2 **Cox regression analysis of PFS and OS for NSCLC patients.** In the Cox regression, IgG responses against 38 and 32 EGFR-derived peptides showed p-values of less than 5% for PFS and OS, respectively. When FDR was controlled at the 5% level, IgG responses against 35 and 20 peptides were identified as significant for PFS and OS, respectively.
(XLSX)
