# Supplementary figures and images for: Humoral Immune Responses to EGFR-Derived Peptides Predict Progression-Free and Overall Survival of Non-Small Cell Lung Cancer Patients Receiving Gefitinib
Source: PLoS One. 2014 Jan 31;9(1):e86667. doi: 10.1371/journal.pone.0086667 (PMC3909003; doi:10.1371/journal.pone.0086667)

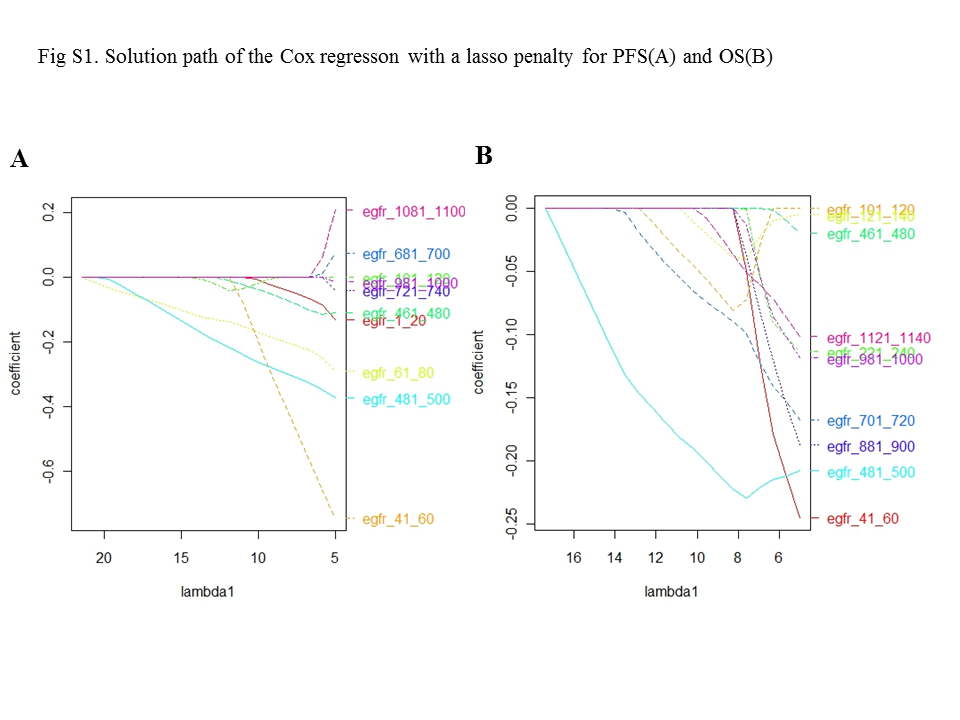

Supplement: Figure S1 — Solution path of the Cox regresson with a lasso penalty for PFS (A) and OS(B). (A)By Cox regression with the lasso penalty, IgG titers against the egfr_41_60, egfr_61_80, and egfr_481_500 peptides had relatively large effects on PFS. (B) By Cox regression with the lasso penalty, IgG titers against the egfr_41_60, egfr_481_500, and egfr_881_900 peptides were shown to have relatively large effects on OS. (TIF) [file pone.0086667.s001.tif]
